# Fucosterol Suppresses the Progression of Human Ovarian Cancer by Inducing Mitochondrial Dysfunction and Endoplasmic Reticulum Stress

**DOI:** 10.3390/md18050261

**Published:** 2020-05-16

**Authors:** Hyocheol Bae, Jin-Young Lee, Gwonhwa Song, Whasun Lim

**Affiliations:** 1 Institute of Animal Molecular Biotechnology, Department of Biotechnology, College of Life Sciences and Biotechnology, Korea University, Seoul 02841, Korea; bhc7@korea.ac.kr; 2 Department of Pharmacology and Toxicology, Medical College of Wisconsin, Milwaukee, Wisconsin, WI 53226, USA; jylee@mcw.edu; 3 Department of Food and Nutrition, College of Science and Technology, Kookmin University, Seoul 02707, Korea

**Keywords:** fucosterol, ovarian cancer, mitochondria, ER, viability

## Abstract

Ovarian cancer is difficult to diagnose early and has high rates of relapse and mortality. Therefore, the treatment of ovarian cancer needs to be improved. Recently, several studies have been conducted in an attempt to develop anticancer drugs from naturally derived ingredients. Compared to traditional chemotherapy, natural compounds can overcome drug resistance with lower side effects. Fucosterol, a phytosterol present in brown algae, reportedly possesses many bioactive effects, including anticancer properties. However, the anticancer effects of fucosterol in ovarian cancer remain unexplored. Therefore, we investigated the effects of fucosterol on progression in human ovarian cancer cells. Fucosterol inhibited cell proliferation and cell-cycle progression in ovarian cancer cells. Additionally, fucosterol regulated the proliferation-related signaling pathways, the production of reactive oxygen species, mitochondrial function, endoplasmic reticulum stress, angiogenesis, and calcium homeostasis. Moreover, it decreased tumor formation in a zebrafish xenograft model. These results indicate that fucosterol could be used as a potential therapeutic agent in ovarian cancer.

## 1. Introduction

Ovarian cancer, a gynecological malignancy, has a high mortality rate [1]. Most ovarian cancers are diagnosed at a later stage of progression. Among patients diagnosed with ovarian cancer, 51% are diagnosed at Stage III and 29% at Stage IV [2]. Despite treatment with a combination of improved surgery, supportive care, and chemotherapeutic drugs, tumors consistently relapse. After treatment, patients with Stage III–IV relapse in 70% of cases, and patients with Stage I–II relapse in 20% [3]. In 1996, combination therapy with paclitaxel and cisplatin was introduced as a standard treatment in ovarian cancer patients [4]. However, the mortality rate for ovarian cancer is still high, and the average five-year survival rate for women with advanced ovarian cancer is lower than 50% [5]. To improve therapeutic outcomes, trials evaluating biological agents in combination with primary chemotherapeutic agents, as well as in maintenance after the completion of chemotherapy are required. 

Seaweed is readily available worldwide and belongs to the marine plant group. Recent studies have focused on the biological and pharmacological activities of seaweeds, as well as their secondary metabolites, for the development of new drugs. Various bioactive compounds, such as phlorotannins, diterpenes, and polysaccharides, produced in seaweed have already been well examined; however, fucosterol, a plant sterol, needs further investigation [6]. Fucosterol (24-ethylidene cholesterol) is a phytosterol extracted from seaweed, algae, and diatoms and has been reported to have several biological activities [7,8]. Notably, the anticancer effects of fucosterol have been studied in various types of cancer, including HT-29 colon cancer cells [9], breast cancer [10,11], promyelocytic leukemia [9], lung cancer [12], and cervical cancer [13]. In terms of the anticancer mechanism of fucosterol, it selectively suppresses HeLa human cervical cancer cells by inducing apoptosis and inhibiting the PI3K/AKT cascade [13]. However, the effects of fucosterol in human ovarian cancer remain unexplored.

Therefore, we investigated the suppressive effect of fucosterol on the progression of ovarian cancer in vitro and in vivo. This study aimed to (1) identify the effects of fucosterol on cell viability and apoptosis in ovarian cancer cells (ES2 and OV90 cells); (2) investigate the regulatory effects of fucosterol on cancer cell progression and the production of reactive oxygen species (ROS) in ES2 and OV90 cells; (3) identify the fucosterol-mediated mitochondrial and endoplasmic reticulum (ER) function in ovarian cancer cells; (4) investigate the synergistic effects of fucosterol in combination with cisplatin or paclitaxel on cell apoptosis and angiogenesis; and (5) evaluate the effects of fucosterol on zebrafish embryos in vivo. Collectively, the present study presents the first evidence of the anticancer effects of fucosterol in human ovarian cancer cells, indicating the possibility of developing this compound as a novel therapeutic agent to inhibit progression in ovarian cancer. 

## 2. Results

### 2.1. Fucosterol Suppressed Cell Growth and Induced Apoptosis in Ovarian Cancer Cells

**Figure 1:**
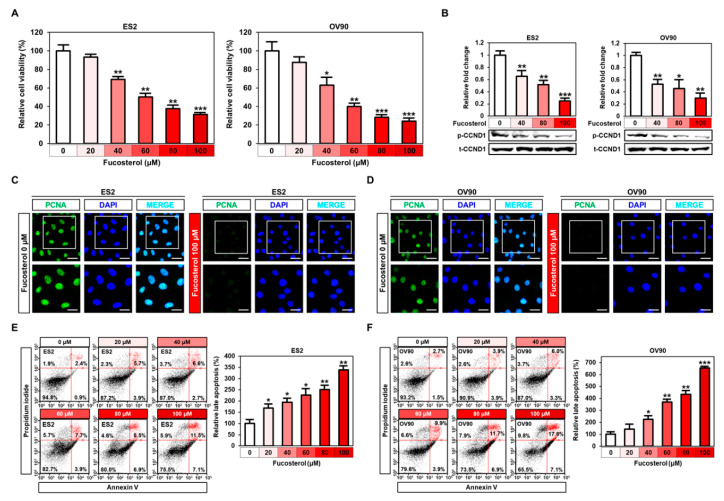
Effects of fucosterol on cell viability and apoptosis in ES2 and OV90 cells. (**A**) Cell proliferation assay using BrdU indicated that fucosterol suppressed ES2 and OV90 cell proliferation in a dose-dependent manner (0, 20, 40, 60, 80, and 100 µM). Data are percentages relative to vehicle-treated control cells (100%). (**B**) Expression of phosphor-CCND1 and total CCND1 proteins induced by fucosterol in ES2 and OV90 cells. (**C**,**D**) Immunofluorescence analysis of PCNA protein in ES2 and OV90 cells. The abundant expression of PCNA protein in the nuclei of ES2 and OV90 cells was reduced following fucosterol treatment in both ovarian cancer cell lines. Scale bar indicates 40 µm (first horizontal panels) and 20 µm (second horizontal panels). (**E**,**F**) Fucosterol induced late apoptosis in ES2 and OV90 cells. Fucosterol increased annexin-V-stained late-apoptotic (upper right quadrant) ES2 and OV90 cells dose-dependently (0, 20, 40, 60, 80, and 100 µM), as demonstrated using flow cytometry. Data are percentages relative to vehicle-treated control cells (100%). Asterisks indicate significant effects compared to the control (* *p* < 0.05, ** *p* < 0.01, and *** *p* < 0.001).

To identify the effect of fucosterol on the viability of both cell lines, we conducted cell proliferation assays using BrdU as an indicator of DNA synthesis in ES2 and OV90 cells. In these cancerous ovarian cells, fucosterol (ranging from 0 to 100 μM) significantly decreased the viability to 31.3% (*p* < 0.001; ES2 cells) and 24.2% (*p* < 0.001; OV90 cells) in a dose-dependent manner, compared to non-treated cells (Figure 1A). Based on these results, the IC_50_ was evaluated to be 62.4 μM in ES2 cells, and 51.4 μM in OV90 cells respectively. In the Western blot analysis, fucosterol (0, 40, 80, and 100 μM) dose-dependently decreased the phosphorylation of cyclin D1 (CCND1), which is associated with cell-cycle regulation (Figure 1B) regulation [14]. PCNA, a well-known representative ovarian cancer marker, was decreased in the nuclei of fucosterol-treated ES2 and OV90 cells compared to vehicle treatment, respectively (Figure 1C,D). Furthermore, the late apoptotic cells were increased in response to fucosterol (0, 20, 40, 60, 80, and 100 μM), as assessed by flow cytometry performed in cells stained with annexin V and propidium iodide (PI) solution. In both ES2 and OV90 cells, apoptosis was induced by fucosterol in a dose-dependent manner, whereas healthy cells were reduced. In the highest concentration of fucosterol, the early apoptotic population was maximally increased 7.89-fold in ES2 cells and 4.73-fold in OV90 cells. For late apoptosis, 4.79-fold in ES2 cells and 6.52-fold in OV90 cells were stimulated by 100 μM fucosterol. In line with apoptotic cells, necrosis was also induced 3.11-fold in ES2 and 3.77-fold in OV90 cells (Figure 1E,F). Consistent with the reduction of cell viability, fucosterol stimulated apoptotic cells in both cell lines. Overall, these results suggest that fucosterol suppresses proliferation and increases apoptosis in human ovarian cancer cells. 

### 2.2. Regulation of Fucosterol on Cell Cycle Progression and ROS Production 

**Figure 2:**
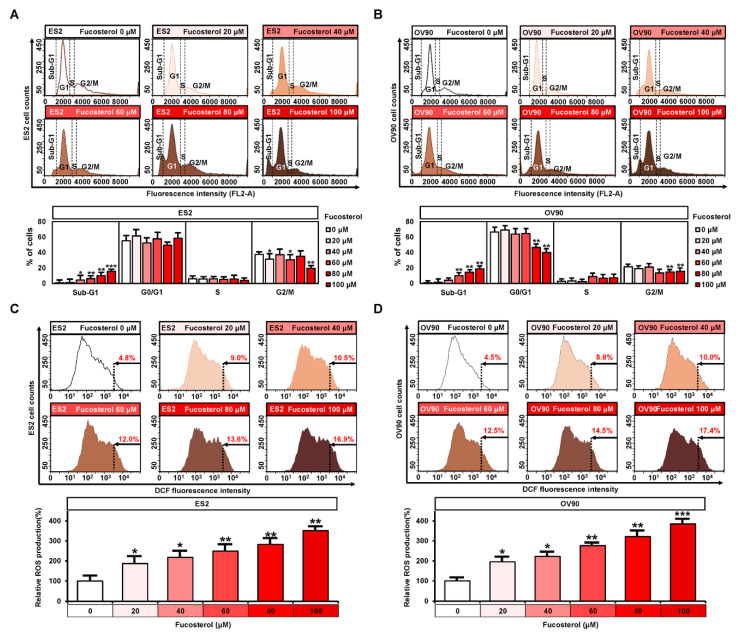
Regulatory effects of fucosterol on cell-cycle progression and reactive oxygen species (ROS) production in ES2 and OV90 cells. (**A**,**B**) Changes in cell-cycle phases were analyzed using flow cytometry with PI-stained ES2 and OV90 cells after incubation with fucosterol. The percentage of fucosterol-treated ovarian cancer cells is indicated at each stage of the cell cycle. (**C**,**D**) ROS production in fucosterol-treated ovarian cancer cells was estimated by dichlorofluorescein (DCF) fluorescence intensity using flow cytometry and compared to vehicle-treated ES2 and OV90 cells. Asterisks indicate significant effects compared to the control (* *p* < 0.05, ** *p* < 0.01, and *** *p* < 0.001).

Cell cycle assays were performed to confirm the status of ES2 and OV90 cells following treatment with various doses of fucosterol. In both cell lines, cell in the sub-G1 stage were increased, from 0.9% to 15.7% in ES2 cells and 1.0% to 19.0% in OV90 cells, following treatment with fucosterol (0, 20, 40, 60, 80, and 100 μM). The G0/G1 stage was decreased from 66.6% to 40.1% by fucosterol in OV90 cells, but a significant change was not observed in OV90 cells. In addition, the G2/M stage was decreased from 37.6% to 19.6% in ES2 cells and from 21.7% to 15.6% in OV90 cells (Figure 2A,B). Furthermore, fucosterol induced ROS generation in ES2 (up to 351.8%, *p* < 0.01) and OV90 (up to 385.1%, *p* < 0.001) cells compared with vehicle-treated ovarian cancer cells, as assessed using flow cytometric analysis (Figure 2C,D).

### 2.3. Effect of Fucosterol on the Concentration of Calcium Ions, Mitochondrial Membrane Potential (MMP), and Apoptotic Proteins in Both Cell Lines

**Figure 3:**
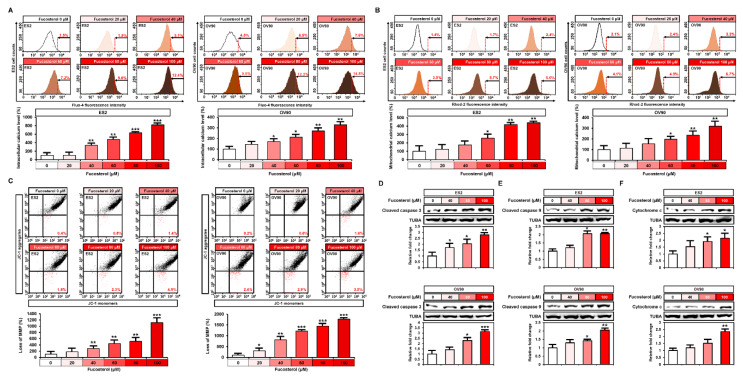
Effects of fucosterol on calcium homeostasis and mitochondrial dysfunction in ES2 and OV90 cells. (**A**) Flow cytometric detection of cytosolic Ca^2+^ following fucosterol treatment (0, 20, 40, 60, 80, and 100 µM) in ES2 and OV90 cells. (**B**) Flow cytometric analysis of mitochondrial Ca^2+^ levels in ES2 and OV90 cells following fucosterol treatment. Dose-dependent effects of fucosterol are represented as a percentage compared to vehicle-treated control values. (**C**) Flow cytometric detection of mitochondrial membrane potential (MMP) in ES2 and OV90 cells treated with fucosterol. Loss of MMP was analyzed using JC-1 red and green fluorescence ratios. (**D**–**F**) Activation of apoptotic proteins such as cleaved caspase 3 (**D**), cleaved caspase 9 (**E**), and cytochrome c (**F**) was analyzed by Western blotting in ES2 and OV90 cells treated with fucosterol. The intensity of the target protein was detected and analyzed relative to that of alpha-tubulin (TUBA) protein. Asterisks indicate significant effects compared to the control (* *p* < 0.05, ** *p* < 0.01, and *** *p* < 0.001).

To investigate the changes in intracellular and mitochondrial calcium levels, human ovarian cancer cells (ES2 and OV90 cells) were stained with Fluo-4 and Rhod-2, respectively, after the treatment with fucosterol, using both for flow cytometry analysis (Figure 3A,B). In both ES2 and OV90 cells, fucosterol dose-dependently induced intracellular calcium concentration up to 818.5% (*p* < 0.001) and 328.1% (*p* < 0.01), respectively, in comparison with vehicle-treated ovarian cancer cells (Figure 3A). Consistently, the mitochondrial calcium concentration was increased in the ES2 (up to 440.1%, *p* < 0.01) and OV90 (up to 319.6%, *p* < 0.01) compared with the control cells (Figure 3B). We then performed JC-1 staining following the fucosterol dose-dependent treatment (0, 20, 40, 60, 80, and 100 μM) of ES2 and OV90 cells to determine whether mitochondrial calcium levels were altered due to fucosterol-disrupted mitochondrial membrane potential (MMP) (Figure 3C). Fucosterol induced the loss of MMP up to 1115.8% (*p* < 0.001) and 1770.0% (*p* < 0.001) in ES2 and OV90 cells, respectively, compared with vehicle-treated control cells. Next, we identified any alterations in the apoptotic protein expression induced by fucosterol (Figure 3D–F). Western blot analysis indicated that fucosterol increased cleaved caspase 3 (ES2: up to 2.7-fold, *p* < 0.01; OV90: up to 3.1-fold, *p* < 0.001), cleaved caspase 9 (ES2: up to 2.0-fold, *p* < 0.01; OV90: up to 2.0-fold, *p* < 0.01), and cytochrome c (ES2: up to 2.1-fold, *p* < 0.05; OV90: up to 2.3-fold, *p* < 0.01) in comparison with the DMSO-treated cells. 

### 2.4. Fucosterol Inactivated PI3K/MAPK Signal Transduction in Ovarian Cancer Cells

**Figure 4:**
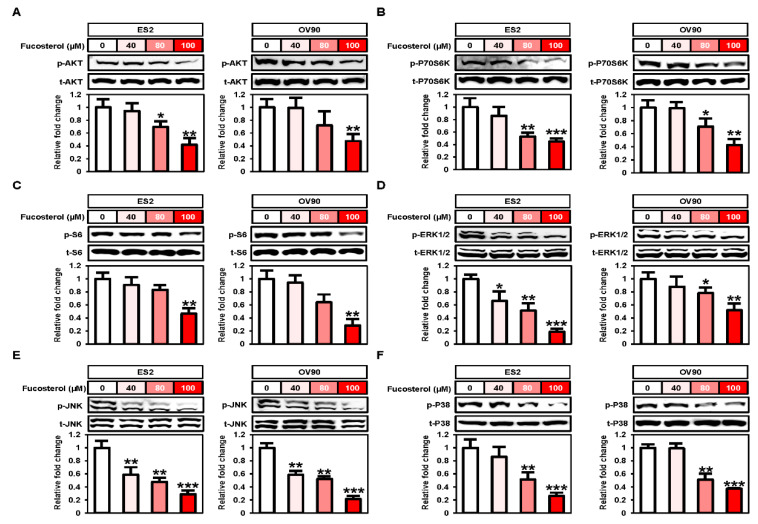
Decrease in PI3K and MAPK signal activities following fucosterol treatment in ovarian cancer cells. (**A**–**F**) Western blot analysis indicated phosphorylated AKT (**A**), P70S6K (**B**), S6 (**C**), ERK1/2 (**D**), JNK (**E**), and P38 (**F**) proteins in fucosterol-treated ES2 and OV90 cells. Values of phosphoprotein intensity were normalized to the intensity of each total protein and are expressed relative to that of the vehicle-treated control cells. Asterisks indicate significant effects compared to the control (* *p* < 0.05, ** *p* < 0.01, and *** *p* < 0.001).

**Figure 5:**
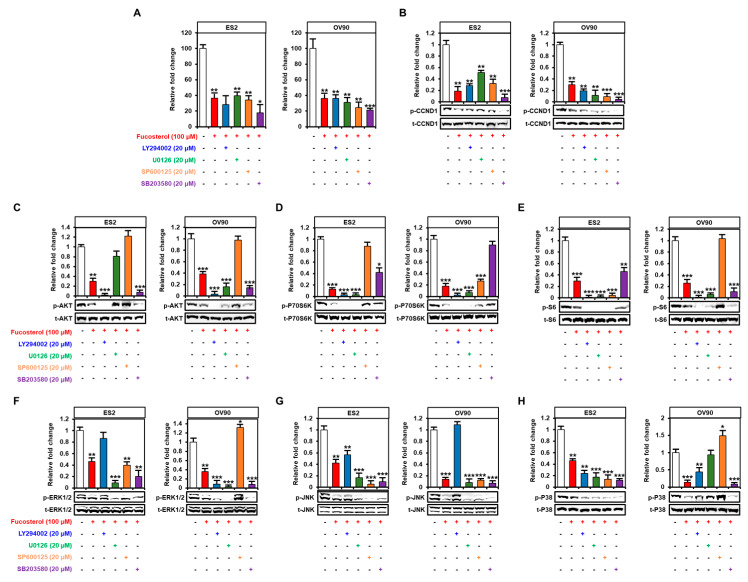
Inhibitory effects of the PI3K and MAPK pathways on the proliferation and phosphorylation of target proteins in ovarian cancer cells. Effects of inhibitors LY294002 (20 μΜ, AKT), U0126 (20 µM, ERK1/2), SP600125 (20 µM, JNK), and SB203580 (20 µM, p38) with fucosterol (100 µM) on cell proliferation (**A**) and phosphorylation of CCND1 (**B**), AKT (**C**), P70S6K (**D**), S6 (**E**), ERK1/2 (**F**), JNK (G), and P38 (**H**) in both ES2 and OV90 cells. Values of phosphoprotein intensity were normalized to the intensity of each total protein and are expressed relative to that of the vehicle-treated control cells. Asterisks indicate significant effects compared to the control (* *p* < 0.05, ** *p* < 0.01, and *** *p* < 0.001).

Next, we performed Western blot analyses to identify the expression of protein kinases belonging to the PI3K and MAPK signal pathways (Figure 4). In ES2 and OV90 cells, phosphorylation of AKT (ES2: up to 0.4-fold, *p* < 0.01; OV90: up to 0.5-fold, *p* < 0.01), P70S6K (up to 0.4-fold, *p* < 0.001; up to 0.4-fold, *p* < 0.01), and S6 (up to 0.5-fold, *p* < 0.01; up to 0.3-fold, *p* < 0.01) decreased gradually following treatment with various doses of fucosterol (0, 40, 80, and 100 μM) (Figure 4A–C). In addition, fucosterol significantly decreased the MAPK pathway phosphorylation cascades, including phosphorylated (p-) ERK1/2 (ES2: up to 0.2-fold, *p* < 0.01; OV90: up to 0.5-fold, *p* < 0.01), p-JNK (ES2: up to 0.3-fold, *p* < 0.001; OV90: up to 0.2-fold, *p* < 0.001), and p-P38 (ES2: up to 0.3-fold, *p* < 0.001; OV90: up to 0.4-fold, *p* < 0.001) in ES2 and OV90 cells compared with vehicle-treated ovarian cancer cells (Figure 4D–F). These results indicated that fucosterol suppressed the PI3K and MAPK signal pathways in ES2 and OV90 cells. In addition, we pretreated with pharmacological inhibitors, including LY294002 (a PI3K inhibitor, 20 µM), U0126 (an ERK1/2 inhibitor, 20 µM), SP600125 (a JNK inhibitor, 20 µM), and SB203580 (a P38 inhibitor, 20 µM), before the treatment with fucosterol in order to identify the fucosterol-associated signal crosstalk in ovarian cancer cells (Figure 5). In both ES2 and OV90 cells, the reduction in cell proliferation was more pronounced following the combination of fucosterol with SB203580 (Figure 5A). p-CCND1 was decreased upon treatment with LY294002, U0126, SP600125, and SB203580 in OV90 cells; however, it was only inhibited by SB203580 in ES2 cells (Figure 5B). Furthermore, fucosterol inhibited the p-AKT without using p-ERK1/2 and p-JNK signaling pathways (Figure 5C). p-P70S6K was also blocked by fucosterol with inhibition of p-AKT and p-EKR1/2, but was not affected by inhibition of the p-JNK and p-P38 pathways (Figure 5D). P-S6 signals were suppressed by fucosterol with inhibition of p-AKT, p-ERK1/2, and p-P38 (Figure 5E). Fucosterol-mediated inhibition of p-ERK1/2 was not affected by inhibition of p-AKT, p-JNK, and p-P38 signals (Figure 5F). These results indicate that p-ERK1/2 signal is regulated by fucosterol upstream of intracellular signal pathways in ovarian cancer cells. In addition, fucosterol inhibited p-JNK with suppression of p-ERK1/2 and p-P38, but the p-AKT pathway did not affect the p-JNK pathway (Figure 5G). Fucosterol-mediated inhibition of p-P38 was not regulated by inhibition of p-AKT, p-ERK1/2, and p-JNK signal pathways (Figure 5H). Overall, it was confirmed that fucosterol inhibits p-ERK1/2 and p-P38 upstream of the signaling mechanism in ovarian cancer, thereby inhibiting the downstream PI3K/MAPK and p-P70S6K signaling mechanisms.

### 2.5. Enhanced Endoplasmic Reticulum (ER) Stress Caused by Fucosterol in Human Ovarian Cancer Cells

**Figure 6:**
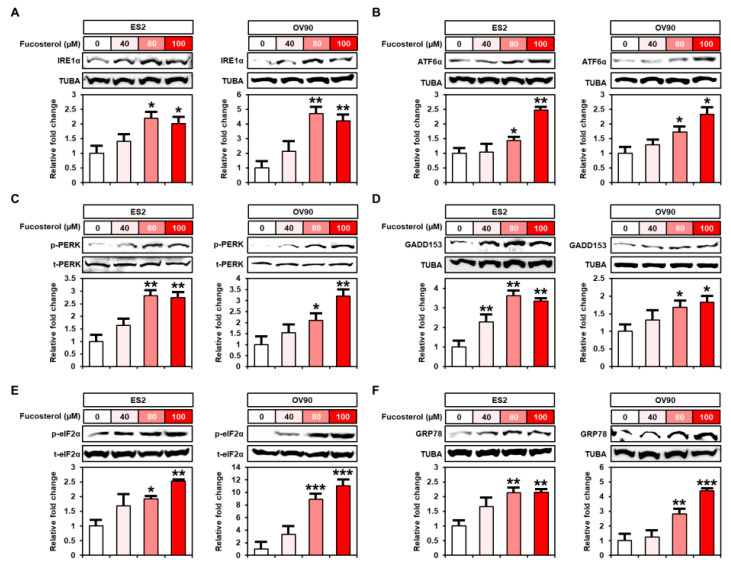
Regulation of fucosterol on ER stress-sensor proteins in ES2 and OV90 cells. (A-F) Activation of ER-stress-associated response genes, such as IRE1α (**A**), ATF6α (**B**), PKR-like ER-resident kinase (PERK) (**C**), GADD153 (**D**), eIF2α (**E**), and GRP78 (**F**), was analyzed using Western blotting in ES2 and OV90 cells treated with various doses of fucosterol (0, 40, 80, and 100 µM). Intensity of the target protein was detected and analyzed relative to total protein or the TUBA protein. Asterisks indicate significant effects compared to the control (* *p* < 0.05, ** *p* < 0.01, and *** *p* < 0.001).

To identify the underlying mechanism of fucosterol on ER stress in ES2 and OV90 cells, we performed Western blot analysis after fucosterol treatment at various doses (0, 40, 80, and 100 μM). In ER-stress-induced cells, the unfolded protein response (UPR) proteins were activated in the ER transmembrane, well-known as ER stress sensors, including inositol-requiring enzyme 1α (IRE1α), activating transcription factor 6α (ATF6α) and PKR-like ER-resident kinase (PERK), growth-arrest- and DNA-damage-inducible gene 153 (GADD153), phosphorylated eukaryotic translation-initiation factor 2α (p-eIF2α), and glucose-regulated protein 78 (GRP78). In ES2 and OV90 cells, Western blot analysis demonstrated that fucosterol increased UPR signaling proteins, including IRE1α (ES2: up to 2.0-fold, *p* < 0.05; OV90: up to 4.2-fold, *p* < 0.01), ATF6α (ES2: up to 2.4-fold, *p* < 0.01; OV90: up to 2.3-fold, *p* < 0.05), p-PERK (ES2: up to 2.7-fold, *p* < 0.01; OV90: up to 3.2-fold, *p* < 0.01), GADD153 (ES2: up to 3.3-fold, *p* < 0.01; OV90: up to 1.8-fold, *p* < 0.05), p-eIF2α (ES2: up 2.5-fold, *p* < 0.01; OV90: up to 11.0-fold, *p* < 0.001), and GRP78 (ES2: up to 2.1-fold, *p* < 0.01; OV90: up to 4.3-fold, *p* < 0.001) compared with DMSO-treated control ovarian cancer cells (Figure 6).

### 2.6. Synergistic Effects of Fucosterol and Cisplatin or Paclitaxel on Apoptotic Signaling Pathways and Anti-Angiogenesis Effects

**Figure 7:**
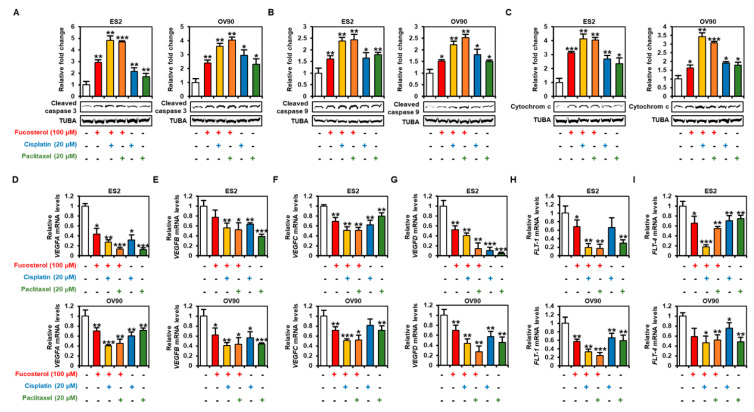
Synergistic effect of fucosterol with cisplatin or paclitaxel on apoptotic proteins and angiogenic genes in ES2 and OV90 cells. (**A**–**C**) Activation of apoptotic proteins, cleaved caspase 3 (**A**), cleaved caspase 9 (**B**), and cytochrome c (**C**), was analyzed using Western blotting in ES2 and OV90 cells treated with a single treatment of fucosterol, cisplatin, or paclitaxel, and combinations thereof. Intensity of the target protein was detected and analyzed relative to the TUBA protein. (**D**–**I**) Expression of the angiogenic related genes, including *VEGFA*, *VEGFB*, *VEGFC*, *VEGFD*, *FLT-1*, and *FLT-4*, was analyzed by quantitative RT-PCR in ES2 and OV90 cells treated with a single treatment of fucosterol, cisplatin, or paclitaxel, and combinations thereof. The asterisks indicate significant differences compared to non-treated cells. Asterisks indicate significant effects compared to the control (* *p* < 0.05, ** *p* < 0.01, and *** *p* < 0.001).

To identify the comparative effects of traditional chemotherapy (cisplatin and paclitaxel) and combined effects of fucosterol with the chemotherapeutic drugs, we performed Western blot analysis using the ovarian cancer cells (Figure 7A–C). In both ES2 and OV90 cells, the cleavage of caspases and release of cytochrome c were increased following fucosterol with cisplatin or paclitaxel co-treatment compared to single treatment with fucosterol, cisplatin, or paclitaxel. Quantitative reverse transcription polymerase chain reaction (RT-PCR) analysis then demonstrated that the *vascular endothelial growth factor A* (*VEGFA*) mRNA expression was reduced by fucosterol. Additionally, co-treatment of fucosterol with cisplatin markedly decreased *VEGFA* expression compared to each treatment in ES2 and OV90 cells (Figure 7D). In addition, the expression of *VEGFB* decreased in fucosterol-treated ovarian cancer cells compared to non-treated cells (Figure 7E). Relative *VEGFC* mRNA levels were inhibited by combined treatment of fucosterol with cisplatin or paclitaxel compared to the single treatment (Figure 7F). Furthermore, in both ovarian cancer cell lines, the reduced expression of *VEGFD* induced by fucosterol was significantly decreased following additional co-treatment with chemotherapeutic agents compared with control cells (Figure 7G). *FLT-1* and *FLT-4* expressions were suppressed following co-treatment of fucosterol with chemotherapy compared to individual chemical treatments (Figure 7H,I). These results indicated that fucosterol combined with chemotherapy enhanced the therapeutic effects by increasing apoptotic effects and anti-angiogenic effects in ovarian cancer cells.

### 2.7. Effects of Fucosterol on Cytotoxicity, Tumorigenesis, and Angiogenesis In Vivo

**Figure 8:**
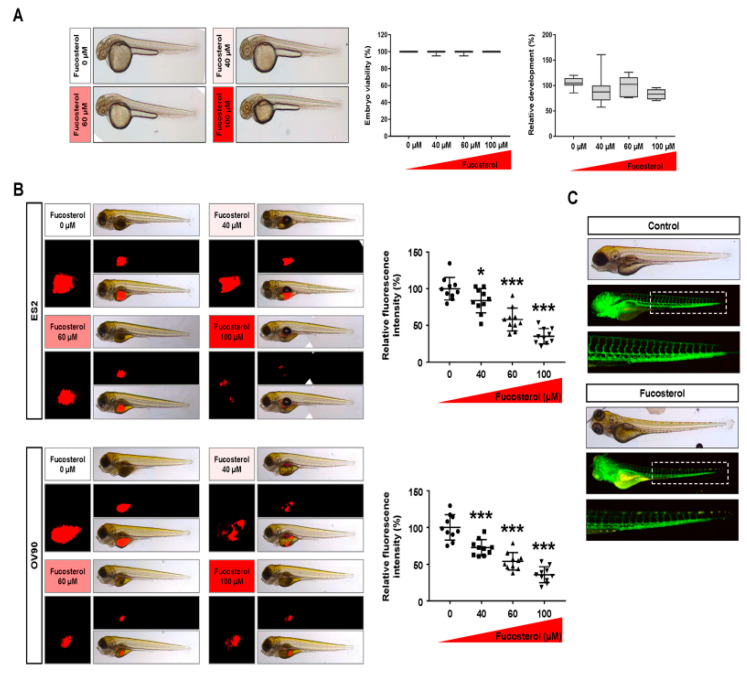
Effects of fucosterol on cytotoxicity, tumorigenesis, and angiogenesis in vivo. (**A**) Zebrafish embryos with the eggshell and pigment removed were treated with various doses of fucosterol for 24 h. Viability and development of normal zebrafish embryos were observed under light microscopy after 24 h of fucosterol treatment. (**B**) Inhibitory effects of fucosterol on cancer development in zebrafish xenograft model. Fucosterol-treated ovarian cancer cells were injected into zebrafish yolks. Zebrafish were incubated in 24 well plates containing Danieau’s solution with 0.003% phenylthiourea at 28.5 °C for 72 h. CM-Dil dye-stained fluorescent tumors were quantified using ImageJ software. (**C**) In vivo validation of fucosterol efficacy in preventing angiogenesis. A zebrafish transgenic model (fli1:EGFP) was treated with fucosterol to determine physiological anti-angiogenic effects of fucosterol. Asterisks indicate significant effects compared to the control (* *p* < 0.05 and *** *p* < 0.001).

Zebrafish embryos were used to examine the cytotoxicity of fucosterol. Zebrafish embryos were extracted from the eggshell and treated with various doses of fucosterol (0, 40, 60, and 100 μM) for 24 h. Notably, embryo viability and development were not affected at all fucosterol concentrations (Figure 8A). Moreover, we confirmed the effects of fucosterol on tumorigenesis using a zebrafish xenograft. In zebrafish yolks, fucosterol inhibited tumor formation in the ovarian cancer cell lines (Figure 8B). The tumor formation was decreased to 58.0% (*p* < 0.001) and 39.4% (*p* < 0.001) in 60 and 100 μM fucosterol-treated ES2 cells, respectively. In OV90 cells, the ratio of tumor formation decreased to 60.4% (*p* < 0.001) and 38.5% (*p* < 0.001) with the 60 and 100 μM fucosterol treatments, respectively. Furthermore, we examined vascular formation in transgenic zebrafish (fli1:EGFP) treated with fucosterol to examine the anti-angiogenic effects in vivo (Figure 8C). The vasculature of the dorsal longitudinal anastomotic vessel, intersegmental vessel, and parts of the dorsal aorta was interrupted in the fli1 transgenic zebrafish embryos following exposure to fucosterol. These results indicated that fucosterol inhibited tumorigenesis and angiogenesis without cytotoxicity in vivo.

## 3. Discussion

Research on the development of novel cancer drugs has significantly increased with the goal to enhance the survival rate in cancer patients. However, owing to the side effects of synthetic drugs and the recurrence of the disease, the development of anticancer drugs from new natural sources such as plants and marine algae is crucial [15]. This is because the side effects associated with natural medicines are less aggressive than those encountered with synthetic chemicals, and are free from resistance to antineoplastic drugs. Combination therapies with natural compounds have been studied for the improvement of chemosensitivity in ovarian cancer [16,17].

Fucosterol has various biological activities, including anticancer, antidiabetic, antioxidant, hepatoprotective, anti-hyperlipidemic, anti-fungal, and blood-cholesterol-reducing activities [7,8]. The cytotoxicity of fucosterol against cancer cells was reported in KB cells (oral epidermoid carcinoma cells), A-549 cells (a human lung tumor line), HT-29 cells (human colon cells [12], HL-60 cells (differentiating myeloid cells) [18], breast carcinoma cell lines [19], WEHI-3 cancer cells (leukemia cells) [20], and cervical cancer [13]. In further detail, fucosterol decreased the development of cancer cells via cell-cycle arrest at the G2/M phase in leukemia and cervical cancer [13,20]. In addition, fucosterol induced death in HeLa cells, stimulated the generation of ROS, and produced mitochondrial dysfunction and pro-apoptotic signals. [18,21]. As previously reported, our results demonstrated that fucosterol increased the sub-G1 phase, ROS generation, and mitochondrial dysfunction, with activation of caspase-3 and -9 and release of cytochrome c, in the ES2 and OV90 cells. In addition, in ES2 and OV90 cells, fucosterol synergistically increased levels of pro-apoptotic proteins in combination with cisplatin or paclitaxel compared to a single treatment. These results support the idea that fucosterol sensitized the drug response from cisplatin and paclitaxel, which means that fucosterol may be able to help to overcome drug resistance in ovarian cancer.

Conventional anticancer agents inhibit the cell proliferation of cancer cells in order to suppress the development of cancer [22,23]. In mammalian cells, the expression of PCNA increases as the cell cycle accelerates. PCNA has proven to be a useful marker for cancer cell proliferation and prognostic evaluation [24]. Chemicals that increase the sub-G1 phase have the possibility of being used as anticancer agents [25]. We confirmed that fucosterol inhibits cell proliferation and PCNA expression in ovarian cancer. Fucosterol also increased the percentage of sub-G1 phase (apoptotic cells) in human ovarian cancer cells. ROS can induce cell apoptosis either directly or through activation of the intracellular pro-apoptotic pathways. Cisplatin induces a mitochondrial-dependent ROS reaction, leading to cytotoxic effects via nDNA and mtDNA damage [26]. Mitochondrial-dependent cell death is associated with mitochondrial calcium concentration overload and MMP, as well as the cleavage of caspases, the release of cytochrome c, and activation of pro-apoptotic proteins [27]. In particular, mitochondrial Ca^2+^ overload can affect the release of pro-apoptotic factors by damaging the mitochondria. In cancer cells, mitochondrial malfunction results in increased cytosolic Ca^2+^ levels by inducing apoptosis [28]. The results of the present study demonstrated that fucosterol stimulated cell death in ovarian cancer cell lines via ROS production and an increase in Ca^2+^ concentration in both the cytoplasm and mitochondria. In general, low levels of pro-apoptotic signals are detected in poor prognosis cancers [29]. Cleaved caspase 3, cleaved caspase 9, and cytochrome c induce programmed cell apoptosis. Paclitaxel causes cell apoptosis through DNA fragmentation, with activation of apoptotic signals including cleaved caspase 3, cleaved caspase 9, and cytochrome c [30]. In our results, fucosterol induced cell apoptotic signals in both cell lines. These intracellular signals are pivotal in cancer development and chemotherapy resistance. In human cervical cancer, the activation of the PI3K/AKT/mTOR pathway, the main system utilized by cancer cells, was reduced following fucosterol treatment [13]. As previously reported, fucosterol dramatically inhibited the phosphorylation of PI3K/MAPK signal transduction in human ovarian cancer cells. The mTOR/p70S6K pathway is activated in ovarian cancer, and carboplatin inhibits the growth of ovarian cancer cells through suppression of mTOR/p70S6K [31]. Activation of the MEK-S6 pathway is also observed in high-grade ovarian cancers [32], and suppression of p-S6 increased the anti-cancer effect of paclitaxel in ovarian cancer cells [33]. In our results, fucosterol inhibited the expression of p-P70S6K and p-S6. Taken together, these results indicate that fucosterol regulates p-ERK1/2 and p-P38 upstream of the ovarian cancer signaling mechanism to suppress the downstream signaling pathways. In addition, mitochondrial dysfunction and the inhibition of the PI3K pathway decrease MMP and increase cytochrome c for apoptosis in cancer cells [34].

ER and angiogenesis are important for the management of cancer progression. First, the ER plays a pivotal role in the synthesis, folding, modification, and transport of proteins [35]. Mitochondria and the endoplasmic reticulum are key organelles in the regulation of cellular homeostasis and energy production through ion exchange with reversible channels. In particular, ER stress induces the unfolded protein response (UPR) and mitochondrial calcium overload, thereby leading to mitochondrial dysfunction and apoptosis [36]. In addition, ER stress induces angiogenic abnormalities via the functional reduction of angiogenic progenitor cells [37], and anti-angiogenic proteins promote protein-trafficking signals to mitochondria, triggering the apoptosis pathway in human umbilical vein endothelial cells (HUVECs) [38,39]. Since mitochondria and the endoplasmic reticulum interact to promote vascular remodeling [40], these key mechanisms have been considered as anticancer targets to attenuate angiogenesis and metastasis in cancer. Therefore, we first proved the anti-angiogenic effect of fucosterol in vivo by using a fli:1 transgenic zebrafish model, generating results beyond the in vitro effect. Fucosterol reduces cognitive dysfunction in aging rats via the downregulation of ER stress. However, the effects of fucosterol on ER stress in cancerous cells have not been elucidated. The present study indicated that fucosterol activated the ER stress sensor proteins, including IRE1α, ATF6α, PERK, GADD153, eIF2α, and GRP78, in the cell death of ES2 and OV90 cells. 

According to the reports for clinical outcomes in ovarian cancer, most chemotherapeutic failures are based on angiogenesis-derived metastasized tumors and ovarian cancer as metastatic disease [41]. Although metastasis contributes to a substantially lower survival rate in ovarian cancers, overcoming metastatic tumors using drug-mediated angiogenesis suppression has yet to be explored. Angiogenesis and the supply of oxygen and nutrients are important for the metastasis of cancerous cells [42]. Thus, the suppression of angiogenesis-related genes is a major target of anticancer therapy. In our results, genes related to vascular development, including *VEGFA*, *VEGFB*, *VEGFC*, *VEGFD*, *FLT-1*, and *FLT-4*, were synergistically decreased by fucosterol in combination with cisplatin or paclitaxel. These results indicate that fucosteol suppresses angiogenesis and metastasis of ovarian cancer. In agreement with the results from angiogenesis-associated genes, vascular development was interrupted in response to fucosterol in the fli1 transgenic zebrafish embryo. Furthermore, fucosterol has no cytotoxicity in normal cell lines including normal colon fibroblast cell [43] and normal breast cell [11], which corresponds with our results that fucosterol had no significant effect on the survival and growth of zebrafish embryos. Fucosterol only selectively inhibited the tumor development of ovarian cancer cells transplanted into zebrafish embryos. Collectively, fucosterol exerts anticancer and anti-angiogenic effects in vivo without cytotoxicity. The pharmacological implications of fucosterol are also supported by its anti-angiogenic function, and it may contribute to enhancing drug sensitivity in metastatic ovarian cancer.

**Figure 9:**
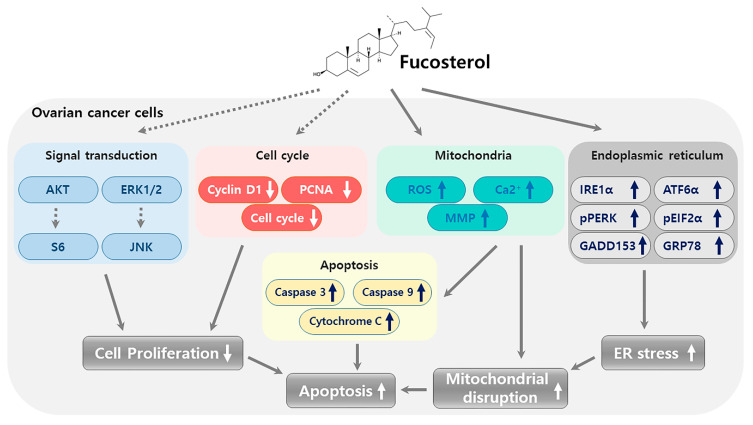
Schematic illustration of fucosterol-triggering anticancer mechanisms in human ovarian cancer cells, which occur via decreasing PCNA expression and cell-cycle regulatory protein expressions in ES2 and OV90 cells.

In conclusion, the present study reported the first evidence of the anticancer effects of fucosterol in human ovarian cancer cells. To provide comprehensive mechanistic insight into the anticancer function of fucosterol in ovarian cancer cells, we provide a graphical illustration in Figure 9 that displays a suggested integration of the obtained results. Fucosterol suppressed cell proliferation by decreasing PCNA expression and cell-cycle regulatory protein expression levels in ES2 and OV90 cells. Fucosterol also increased apoptosis via caspase 3, 9, and cytochrome c activation, which was mainly due to mitochondrial dysfunction. The depolarization of the MMP and the production of ROS, increasing the calcium concentration support the integrated loss of mitochondrial function mediated apoptosis induction and cell-cycle arrest. Following the mitochondrial stress, ER stress was also increased by fucosterol via regulation of ER-associated protein expression. This implies that the fucosterol-mediated anticancer effect is based on an interaction of concomitant cellular stresses in ovarian cancer cells which contributes to the synergistic anticancer effect in combination with cisplatin and paclitaxel. In intracellular mechanisms, fucosterol inhibited signal transduction pathways, including PI3K and MAPK in both cell lines. In in vivo experiments, fucosterol selectively inhibited ovarian cancer growth in zebrafish xenografts without embryo cytotoxicity. Additionally, vascular formation was suppressed by fucosterol, demonstrated in a fli:1 transgenic zebrafish model and via reduction of angiogenesis-regulatory gene expression. These results indicate that fucosterol induced apoptotic mechanisms to suppress the progression of human ovarian cancer cells through mitochondrial dysfunction, ER stress, and inhibition of angiogenesis in ES2 and OV90 cells.

## 4. Materials and Methods 

### 4.1. Reagents

Fucosterol (catalog number: 21858) was purchased from the Cayman Chemical Company (Ann Arbor, MI, USA) and dissolved in dimethyl sulfoxide (DMSO) before use. The antibodies used in this study are listed in Table 1.

### 4.2. Cell Culture

ES2 and OV90 cell lines were purchased from the American Type Culture Collection (Manassas, VA, USA) and maintained as previously described [44].

### 4.3. Proliferation Assay

Cell Proliferation ELISA, BrdU Kit (Roche, Basel, Switzerland), was used for to evaluate cell growth. Briefly, the ES2 and OV90 cells were seeded in 96 well plates and then incubated for 24 h in serum-free 5A McCoy’s medium. Cells were then treated with various concentrations of fucosterol to a final volume of 100 µL/well. After 48 h of incubation, 10 mM BrdU was added to the cell culture and the cells were incubated for an additional 2 h at 37 °C.

### 4.4. Immunofluorescence Microscopy

The effects of fucosterol on the expression of proliferating-cell nuclear antigen (PCNA) were determined using immunofluorescence microscopy. Briefly, ES2 and OV90 cells were incubated with or without fucosterol (100 µM) at 37 °C for 48 h in a CO_2_ incubator and probed with mouse anti-human monoclonal PCNA at a final dilution of 1:100 (2 µg/mL). Next, the cells were incubated with goat anti-mouse IgG Alexa 488 at a dilution of 1:200 for 1 h at room temperature. The cells were then washed using 0.1% bovine serum albumin (BSA) in phosphate-buffered saline (PBS) and overlaid with 4′,6-diamidino-2-phenylindole (DAPI). For the primary antibody, the images were captured using an LSM710 (Carl Zeiss, Oberkochen, Germany) confocal microscope.

### 4.5. Annexin V and PI Staining

Fucosterol-induced ovarian cancer cell (ES2 and OV90) apoptosis was analyzed using the FITC Annexin V apoptosis detection kit I (BD Biosciences, Franklin Lakes, NJ, USA). Briefly, cells (5 × 10^5^ cells) were seeded on 6 well plates and treated with fucosterol at different doses of 0, 20, 40, 60, 80, and 100 μM for 48 h at 37 °C in a CO_2_ incubator. The supernatants were removed from the culture dishes and the adherent cells were detached with trypsin-EDTA. The cells were collected by centrifugation, washed with PBS, and resuspended using 1× binding buffer at a dilution of 1 × 10^6^ cells/mL. Next, 100 μL of the cell suspension (1 × 10^6^ cells) was transferred to a 5 mL culture tube and incubated with 5 μL FITC Annexin V and 5 μL PI for 15 min at room temperature in the dark. After this, 400 μL of 1× binding buffer was added to a 5 mL culture tube. The fluorescence intensity was determined using the FACS Calibur (BD Biosciences).

### 4.6. Cell-Cycle Assay

In ES2 and OV90 cells, fucosterol’s effects on the cell-cycle stages were evaluated using PI. The cells (5 × 10^5^ cells) were seeded in 6 well plates and treated with fucosterol in a dose-dependent manner for 48 h at 37 °C in a CO_2_ incubator. The supernatants were removed from the culture dishes and the adherent cells were detached with trypsin-ethylenediaminetetraacetic acid (EDTA). The cells were collected by centrifugation, washed with PBS, and resuspended using 1 × binding buffer at a dilution of 1 × 10^6^ cells/mL. Next, 100 μL of the cell suspension (1 × 10^6^ cells) was transferred to a 5 mL culture tube and incubated with 5 μL of RNase A and 5 μL of PI for 30 min at room temperature in the dark. Subsequently, 300 μL of 1× binding buffer was added to a 5 mL FACS tube. The fluorescence intensity was determined using FACSCalibur (BD Biosciences).

### 4.7. Determination of Cellular ROS

Intracellular ROS production was estimated using 2′,7′-dichlorofluorescin diacetate (Sigma, St. Louis, MO, USA), which is converted to fluorescent 2′,7′-dichlorofluorescin (DCF) in the presence of peroxides. ES2 and OV90 cells were detached with trypsin-EDTA, collected by centrifugation, and washed with PBS. The cells were treated with 10 µM DCFH-DA for 30 min at 37 °C. The cells were then washed twice with PBS and treated with different doses of fucosterol for 1 h at 37 °C in a CO_2_ incubator. Next, the treated cells were washed with PBS again. Fluorescent DCF intensity was analyzed using a flow cytometer (BD Bioscience).

### 4.8. Measurement of Concentrations of Cytosol Ca^2+^

ES2 and OV90 cells were seeded on 6 well plates and incubated for 24 h in serum-free medium when the cells attained 70%-80% confluency. The cells were then treated with different concentrations of fucosterol for 48 h at 37 °C in a CO_2_ incubator. Supernatants were removed from the culture dishes and adherent cells were detached with trypsin-EDTA. The cells were collected by centrifugation. Collected cells were resuspended using 3 μM Fluo-4 and incubated at 37 °C in a CO_2_ incubator for 20 min. The stained cells were washed with PBS and fluorescent intensity was analyzed using a flow cytometer (BD Bioscience).

### 4.9. Measurement of Mitochondrial Ca^2+^ Concentration

ES2 and OV90 cells were seeded on 6 well plates and incubated for 24 h in the serum-free medium when the cells attained 70%–80% confluency. Next, the cells were treated with different concentrations of fucosterol for 48 h at 37 °C in a CO_2_ incubator. Supernatants were removed from the culture dishes and adherent cells were detached using trypsin-EDTA. The cells were collected by centrifugation, with the collected cells resuspended using 3 μM Rhod-2 and incubated at 37 °C in a CO_2_ incubator for 20 min. The stained cells were washed with PBS. The fluorescent intensity was analyzed using a flow cytometer (BD Bioscience).

### 4.10. JC-1 Mitochondrial Membrane Potential (MMP) Assay

Changes in the JC-1 MMP were determined using a mitochondrial staining kit (Sigma). ES2 and OV90 cells were seeded on 6 well plates and incubated for 24 h in the serum-free medium until 70% confluency was attained. Cells were then treated with fucosterol at various doses for 48 h at 37 °C in a CO_2_ incubator. Supernatants were removed from the culture dishes, and adherent cells were detached using trypsin-EDTA. The cells were collected by centrifugation. Collected cells were resuspended in a staining solution, which included 200 × JC-1 and 1 × staining buffer, and incubated at 37 °C in a CO_2_ incubator for 20 min. The stained cells were collected by centrifugation and washed once with 1 × JC-1 staining buffer. After washing, the cells were centrifuged once more and resuspended in 1 mL staining buffer. The fluorescence intensity was analyzed using FACSCalibur (BD Biosciences).

### 4.11. Western Blot Analysis

The protein concentrations in whole-cell extracts were determined using the Bradford protein assay (Bio-Rad, Hercules, CA, USA) with BSA as the standard. Proteins were denatured, separated by sodium dodecyl sulfate-polyacrylamide gel electrophoresis (SDS-PAGE), and then transferred to nitrocellulose membranes. Blots were developed using enhanced chemiluminescence detection (SuperSignal West Pico, Pierce, Rockford, IL, USA) and quantified by measuring the intensity of light emitted from correctly sized bands under ultraviolet light using a ChemiDoc EQ system and Quantity One software (Bio-Rad). Immunoreactive proteins were detected using goat anti-rabbit polyclonal antibodies against phosphorylated and total proteins at a 1:1000 dilution and separated using 10% SDS-PAGE. As a loading control, total protein and α-tubulin (TUBA) were used to normalize results for the detection of target proteins. Multiple exposures of each Western blot were used to ensure the linearity of chemiluminescent signals.

### 4.12. RNA Isolation

Total cellular RNA was isolated from ovarian cancer cells (ES2 and OV90) using the Trizol reagent (Invitrogen, Carlsbad, CA, USA) and purified using an RNeasy Mini Kit (Qiagen, Hilden, Germany) according to the manufacturer’s guidelines. The quantity and quality of total RNA were determined using spectrometry and denaturing agarose gel electrophoresis, respectively.

### 4.13. Quantitative PCR Analysis

Specific primers for human VEGFA, VEGFB, VEGFC, VEGFD, FLT-1, and FLT-4 were designed based on sequences in the GenBank database using Primer 3 (ver. 4.0.0). All primers were synthesized by Bioneer (Daejeon, Republic of Korea). Gene expression levels were measured using SYBR Green (Sigma) and a StepOnePlus Real-Time PCR System (Applied Biosystems, Waltham, MA, USA). The PCR conditions were 95 °C for 3 min, followed by 40 cycles at 95 °C for 20 sec, 64 °C for 40 sec, and 72 °C for 1 min using a melting curve program (increasing the temperature from 55 to 95 °C at 0.5 °C per 10 sec) and continuous fluorescence measurements. Sequence-specific products were identified by generating a melting curve in which the CT value represented the cycle number at which a fluorescent signal was significantly greater than the background, and the relative gene expression was quantified using the 2-ΔΔCT method. The glyceraldehyde-3-phosphate dehydrogenase (GAPDH) gene was used as the endogenous control to standardize the amount of RNA in each reaction.

### 4.14. Toxicity and Angiogenesis Analysis In Vivo

To confirm the toxicity of fucosterol, zebrafish embryos were treated with fucosterol at various doses. In brief, embryos were exposed to 0.003% phenylthiourea (PTU) for 14 h before fucosterol treatment to suppress pigmentation. The embryo shell was removed and then treated with various doses of fucosterol in 24 well plates for 24 h. After 24 h of treatment, viability and development were observed under light microscopy (Carl Zeiss). Pictures were obtained by fixing zebrafish embryos onto a glass slide with 3% methylcellulose (Sigma). For the angiogenesis assay, transgenic Fli1:GFP larvae were treated with fucosterol for 48 h, and then GFP expression patterns of 10 zebrafish larvae per condition were evaluated. Pictures were obtained using a fluorescence microscope (Leica, Wetzlar, Germany).

### 4.15. Xenografts

Xenograft studies were performed as previously described with modifications [45]. Fertilized eggs were treated with Danieau’s solution containing 0.003% PTU at 28.5 °C for 48 h to suppress pigmentation. Micropipettes were manufactured from a 1.0 mm glass capillary (World Precision Instruments, Sarasota, FL, USA) using a micropipette puller (Shutter Instrument, Novato, CA, USA) for injection and anesthesia. Briefly, 48 h post-fertilization (hpf), zebrafish were anesthetized in 0.02% tricaine (Sigma) and immobilized on an agar plate. Cells were treated with various doses of fucosterol for 22 h; cells were then stained for an additional 2 h with the cell tracker CM-Dil dye (4 μM; Invitrogen). Next, 100–200 cells were injected into the yolk sac by microinjection (PV820 microinjector, World Precision Instruments). Subsequently, the zebrafish were incubated in 24 well plates containing Danieau’s solution with 0.003% PTU at 28.5 °C for 72 h. The fish were immobilized in a drop of 3% methylcellulose in Danieau’s solution on a glass slide. Pictures were obtained using fluorescence microscopy (Leica). Fluorescent tumors were quantified by ImageJ software (U.S. National Institute of Health, Bethesda, MD, USA). 

### 4.16. Statistical Analyses

All quantitative data were subjected to least-squares analysis of variance (ANOVA) using the general linear model procedures of the Statistical Analysis System (SAS Institute Inc. Cary, NC, USA). Western blot data were corrected for differences in the sample loading using total protein or TUBA data as a covariate. All tests of significance were performed using the appropriate error terms corresponding to the expected value to the mean squares for error. A *p*-value less than or equal to 0.05 was considered significant. Data are presented as least-square means (LSMs) with standard errors [46].

## Figures and Tables

**Table 1:** List of antibodies.

Primary Antibodies	Dilution	Supplier	Catalog Number
Phospho-Cyclin D1 (Thr^286^)	1:1000	Cell Signaling	3300
Cyclin D1	1:1000	Cell Signaling	2922
PCNA	1:100	Santa Cruz	sc-56
Phospho-AKT (SER^473^)	1:1000	Cell Signaling	4060
AKT	1:1000	Cell Signaling	9272
Phospho-P70S6K (Thr^421^/Ser^424^)	1:1000	Cell Signaling	9204
P70S6K	1:1000	Cell Signaling	9202
Phospho-S6 (Ser^235/236^)	1:1000	Cell Signaling	2211
S6	1:1000	Cell Signaling	2217
Phospho-ERK1/2(Thr^202^/Tyr^204^)	1:1000	Cell Signaling	9101
ERK1/2	1:1000	Cell Signaling	4695
Phospho-JNK (Thr^183^/Tyr^185^)	1:1000	Cell Signaling	4668
JNK	1:1000	Cell Signaling	9252
Phospho-P38 (Thr^180^/Tyr^182^)	1:1000	Cell Signaling	4511
P38	1:1000	Cell Signaling	9212
IRE1α	1:1000	Cell Signaling	3294
ATF6α	1:1000	Santa Cruz	sc-166659
Phospho-PERK (Thr^981^)	1:1000	Santa Cruz	sc-32577
PERK	1:1000	Santa Cruz	sc-13073
GADD153	1:1000	Santa Cruz	sc-7351
Phospho-eIF2α (Ser^51^)	1:1000	Cell Signaling	3398
eIF2α	1:1000	Cell Signaling	5324
GRP78	1:1000	Santa Cruz	sc-13968
Cleaved caspase-3	1:1000	Cell Signaling	9664
Cleaved caspase-9	1:1000	Cell Signaling	9501
Cytochrome c	1:1000	Cell Signaling	11940
TUBA	1:1000	Santa Cruz	sc-5286
